# A Deep Dive Into the Potential Antiaging Effects of Colla Corii Asini on the Skin: A Comparative Study

**DOI:** 10.1111/jocd.70080

**Published:** 2025-02-27

**Authors:** Fan Wu, Xiao Xie, Wenwen Ru, Xiaofei Guo, Lingfeng Meng, Jianling Zhang, Xiuling Liu, Yao Pan

**Affiliations:** ^1^ Department of Cosmetics, School of Light Industry and Engineering Beijing Technology and Business University Beijing China; ^2^ Beijing Key Laboratory of Plant Resources Research and Development Beijing China; ^3^ National Engineering Research Center for Gelatin‐Based Traditional Chinese Medicine Dong‐E‐E‐Jiao Co. Ltd. Liaocheng Shandong China; ^4^ Liaocheng Functional Food Research and Development Key Laboratory Liaocheng Shandong China

**Keywords:** antiwrinkle tight, Ejiao, in vitro test, moisturizing, repair, skin barrier

## Abstract

**Background:**

Skin aging results in skin barrier damage and skin wrinkles. Oral *Colla Corii Asini* (CCA) can show significant antiaging effects. However, few studies have investigated the skin's antiaging effects of topical CCA. Here, we investigated and compared the effects of CCA crushed (CCAC) and its enzymatic digest, CCA oligopeptide (CCAO), on skin barrier function and wrinkle formation.

**Methods:**

CCAO and CCAC were prepared by enzymatic digestion and pulverization. HaCaT cells and HSF cells were selected as in vitro cell models. Cytotoxicity and cell proliferation were detected by MTT assay; scratch assay was used to evaluate the effect on cell migration; quantitative real‐time PCR (qRT‐PCR), immunofluorescence, and ELISA were used to determine the changes in expression of the barrier‐related molecules (AQP3, FLG, and LOR) and antiwrinkle firming‐related molecules (COL‐1, COL‐3, ELN, and MMP‐1).

**Results:**

MTT assay showed no significant cytotoxicity of CCAO and CCAC at < 2.5 mg/mL. After treating the cells with safe doses of CCAO and CCAC, HaCaT cell proliferation and migration were significantly enhanced, and there was no difference between the two effects. The expression levels of AQP3, FLG, and LOR were all significantly increased compared with the NC group, in which CCAO mainly promoted FLG and LOR expression, while CCAC mainly promoted the expression of AQP3. At the same time, CCAO and CCAC also significantly upregulated the expression levels of COL‐1, COL‐3 and ELN, and downregulated MMP‐1 expression in HSF cells, and the effect of CCAO was more significant.

**Conclusion:**

Topical application of CCA improves the skin barrier, increases collagen levels, and enhances skin elasticity, thus slowing down skin aging. CCAO is more easily absorbed and utilized by the skin due to its small molecular weight, which gives a better effect.

## 1 Introduction

With increasing age, skin morphology and physiology deteriorate, which is the first obvious indicator of the aging process. In today's society, healthy aging is a goal for almost everyone, catalyzing a blossoming and growing antiaging market [1, 2].

Many traditional Chinese medicines have demonstrated strong antiaging properties and can be effectively used in food and cosmetics to delay skin aging [3, 4]. *Colla Corii Asini* (CCA), a traditional Chinese medicine with food and medicine origins, is a solid gelatin made from the dried or fresh skin of donkeys (family Equidae) by decoction and concentration and is mostly used internally. According to the ancient Chinese herbology books “Shennong Bencaojing” and “The Compendium of Materia Medica”, oral CCA has significant antiaging effects [5, 6, 7]. This is because CCA, which is a mixture of peptides and proteins, is digested to form enzyme‐digested CCA (CCAD), the main component of which is a bioactive peptide [8, 9]. The most widely studied bioactive peptides of marine origin, such as fish collagen, have a variety of biological effects, including anti‐inflammatory, antioxidant, antiaging, and wound repair effects [10, 11]. Oral administration of bioactive peptides has also been shown to improve skin condition and skin barrier function [12, 13, 14].

Therefore, CCA is a traditional Chinese medicine with significant antiaging effects that influence human health. However, current studies on CCA have focused primarily on exploring the effects of orally administered CCAD on skin aging. Few studies have focused on the effects of the topical application of CCA and CCAD on skin aging, and no studies have reported the differences between CCAD and CCA in terms of their antiaging effects on skin. The present study accurately and comprehensively evaluated and compared the safety and efficacy of CCA oligopeptide (CCAO) and crushed CCA (CCAC) for the prevention of skin aging.

## 2 Materials and Methods

### 2.1 Preparation of CCAO and CCAC

The CCA was crushed and filtered through a 120‐mesh sieve to make the CCAC.

CCA was crushed and dissolved in distilled water, maintained at 50°C–55°C. It was then digested by proteases (papain, pepsin, and bromelain) at pH 6.5 and 45°C for 2 h. The mixture was filtered through a 120‐mesh sieve to produce CCAO. The peptide sample was then concentrated, vacuum‐dried, and stored at 20°C in a dry environment. CCAO contains more low molecular weight compounds than CCAC.

### 2.2 Cell Culture and Reagents

Human Skin Fibroblasts (HSF) cells and human immortalized epidermal (HaCaT) cells were purchased from Cell Resource Center, IBMS, CAMS/PUMC (Beijing, China), and cultured in DMEM medium containing 10% fetal bovine serum (FBS) and 1% penicillin/streptomycin. The cultures were kept at 37°C in a humidified environment with 5% CO_2_ and 95% air. All cell cultures and phosphate‐buffered saline (PBS) supplements were purchased from Wisent Biotechnology Co.Ltd. (Nanjing, China). CCA (crushed) (CCAC) and CCA oligopeptide (CCAO) were obtained from Dong‐E‐E‐Jiao Co. Ltd. (Shandong, China). AQP3, FLG, COL‐1, HO‐1, β‐actin, and Goat Anti‐Rabbit IgG H&L (Alexa Fluor 488) were purchased from Abcam (Cambridge, UK).

### 2.3 Cell Viability Assay

Cytotoxicity was assessed via the MTT method. HaCaT cells (1 × 10^4^ cells/well) and HSF (1 × 10^4^ cells/well) cells were seeded in 96‐well plates and incubated at 37°C for 24 h. After the medium was removed, the cells were treated with CCAO and CCAC at concentrations ranging from 0.16 to 20.00 mg/mL for an additional 24 h. Each well received 10 μL of MTT solution (5 mg/mL) and was incubated at 37°C for 4 h. After incubation, 100 μL of lysis solution was added to dissolve the formazan crystals. The absorbance at 570 nm was then measured via a Tecan Infinite M200 Pro multimode microplate reader (Tecan Trading AG, Switzerland).

### 2.4 Cell Proliferation Assay

HaCaT cells were seeded in 96‐well plates at a density of 1 × 10^4^ cells/well 1 day before the medium was replaced with serum‐free DMEM containing varying concentrations of CCAO and CCAC. After 24 h, cell viability was assayed via MTT, as described previously.

### 2.5 Cell Migration Assay

Cell migration was evaluated via scratch assays. HaCaT cells (3.5 × 10^5^ cells/well) were seeded in 6‐well plates. The following day, a 200‐μl sterile pipette tip was used to create a scratch. After washing with PBS to remove dislodged cells, fresh complete culture media with varying concentrations of CCAO and CCAC were added. The cells were then starved in serum‐free medium for 24 h. Images of the same area were captured for each treatment condition at 0 and 24 h. The images were analyzed via an inverted microscope to determine the wound healing rate (% of control). The experiments were performed in triplicate.

### 2.6 Real‐Time PCR Measurement

Total RNA was extracted with TRIzol reagent (TransGen Biotech, Beijing, China), after which cDNA was synthesized with the ReverTra Ace qPCR RT kit (TOYOBO, Shanghai, China) following the manufacturer's protocol. RT–PCR was conducted on a CFX96 Real‐time System (LightCycler480 II, Roche, Switzerland) with SYBR Green Real‐time PCR Master Mix (TOYOBO, Shanghai, China). The primers, provided in Table 1, were sourced from AuGCT DNA‐SYN Biotechnology (Beijing, China). Relative gene expression was calculated via the delta–delta Ct method (threshold cycle) on the basis of the endogenous GAPDH loading control normalization.

**TABLE 1:** Real‐time fluorescent quantitative PCR primer sequence.

Primer	Sequence 5′–3′
FLG‐F	GTTACAATTCCAATCCTGTTGTTTTC
FLG‐R	CGTTGCATAATACCTTGGATGATC
LOR‐F	GGCTGCATCTAGTTCTGCTGTTTA
LOR‐R	CAAATTTATTGACTGAGGCACTGG
AQP3‐F	GCTGTCACTCTGGGCATCCTC
AQP3‐R	GCGTCTGTGCCAGGGTGTAG
COL‐1A1‐F	GAGGGCCAAGACGAAGACATC
COL‐1A1‐R	CAGATCACGTCATCGCACAAC
COL‐3A1‐F	GGAGCTGGCTACTTCTCGC
COL‐3A1‐R	GGGAACATCCTCCTTCAACAG
ELN‐F	GCAGGAGTTAAGCCCAAGG
ELN‐R	TGTAGGGCAGTCCATAGCCA
MMP‐1‐F	AAAATTACACGCCAGATTTGCC
MMP‐1‐R	GGTGTGACATTACTCCAGAGTTG
GAPDH‐F	GGAGCGAGATCCCTCCAAAAT
GAPDH‐R	GGCTGTTGTCATACTTCTCATGG

### 2.7 Immunofluorescence Assay

The cells were washed with PBS and then fixed with 4% paraformaldehyde for 15 min. The cells were then washed three times with PBS (5 min each wash) and permeabilized with 0.3% Triton X‐100 in PBS for 10 min. After three additional washes, the cells were blocked with 1% BSA in PBS for 2 h at RT and incubated with primary antibodies overnight at 4°C. The cells were washed 3 times with PBS and then incubated for 2 h at RT with a secondary antibody conjugated to Alexa488. DAPI was used to label the nuclei, and images were obtained with an inverted microscope (magnification, ×40; BX51; Olympus Corporation, Tokyo, Japan).

### 2.8 Quantification of MMP‐1

After 24 h of sample treatment, the supernatants from the cell cultures were collected. MMP‐1 secretion was measured via ELISA kits (Laizi Biotechnology) following the manufacturer's instructions. Each experiment was repeated three times.

### 2.9 Statistical Analysis

Statistical analysis was performed via SPSS software version 25 and GraphPad Prism software. The data are shown as the mean ± standard deviation (SD). Two‐way ANOVA was used to compare the differences between two groups, whereas one‐way ANOVA followed by Dunnett's post hoc test was used to compare multiple treatment groups with the control group. All experiments were conducted in triplicate, and the results are expressed as the means ± SD of three individual experiments. Differences were considered statistically significant when *p* < 0.05.

## 3 Results

### 3.1 Impact of CCAO and CCAC on HSF and HaCaT Cell Viability

**FIGURE 1:**
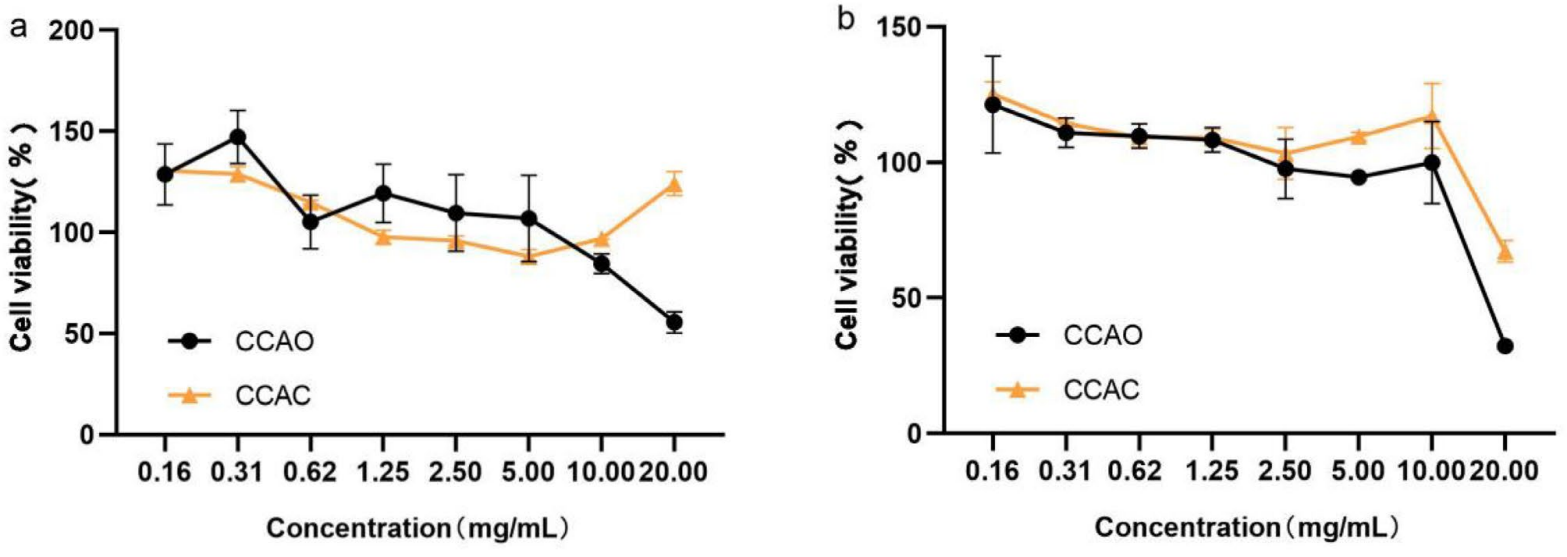
Effect of CCAC and CCAO on the viability of HaCaT and HSF cells was evaluated using the MTT assay. (a) HaCaT cells were treated with CCAC and CCAO (0.16–20.00 mg/mL, 24 h). (b) HSF cells were treated with CCAC and CCAO (0.16–20.00 mg/mL, 24 h).

As shown in Figure 1, the viability of HaCaT cells and HSF cells was not affected by CCAO or CCAC (0.16 *~* 2.500 mg/mL). To investigate the health benefits of CCAO and CCAC, doses of 0.16, 0.62, and 2.50 mg/mL for low, medium, and high concentrations of CCAO and CCAC, respectively, were chosen for the following experiments.

### 3.2 Cell Proliferation

**FIGURE 2:**
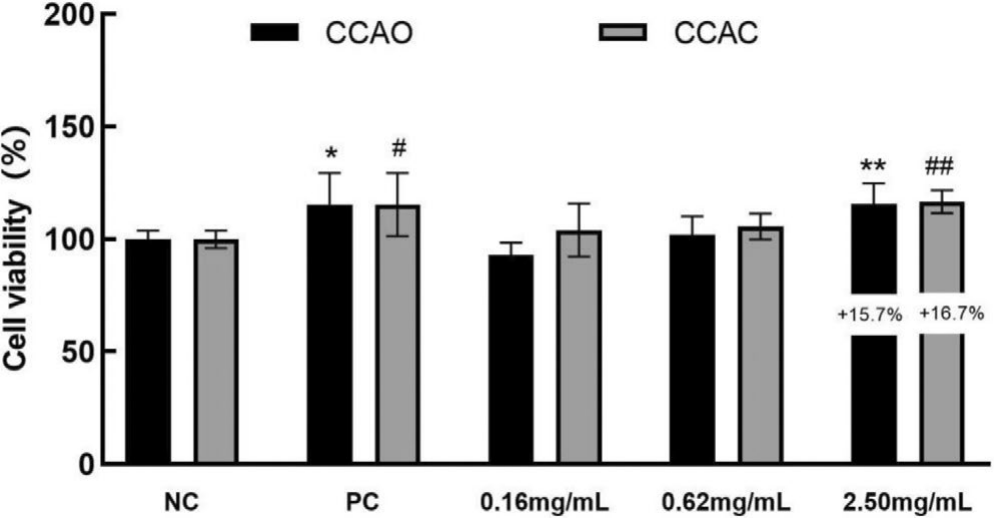
Effects of CCAC and CCAO on HaCaT cell proliferation. An MTT assay was carried out to assess cell proliferation. Complete medium was used as a positive control; HaCaT cells were treated with CCAC or CCAO (0.16, 0.62, or 2.50 mg/mL, 24 h), and the cell proliferation rate was calculated. The data represent three independent experiments, and the results are presented as the means ± SD. *p < 0.05, **p < 0.01 compared with the control under the CCAO treatment condition; #*p* < 0.05, ##*p* < 0.01 compared with the control under the CCAC treatment.

As shown in Figure 2, compared with the NC group, there was no significant difference in the growth of HaCaT cells between the groups stimulated with 0.16 and 0.62 mg/mL CCAO and CCAC. The group stimulated with 2.50 mg/mL had significantly increased HaCaT cell proliferation, with increases of 16.67% and 25.71%, respectively (*p* < 0.05).

### 3.3 Cell Migration

**FIGURE 3:**
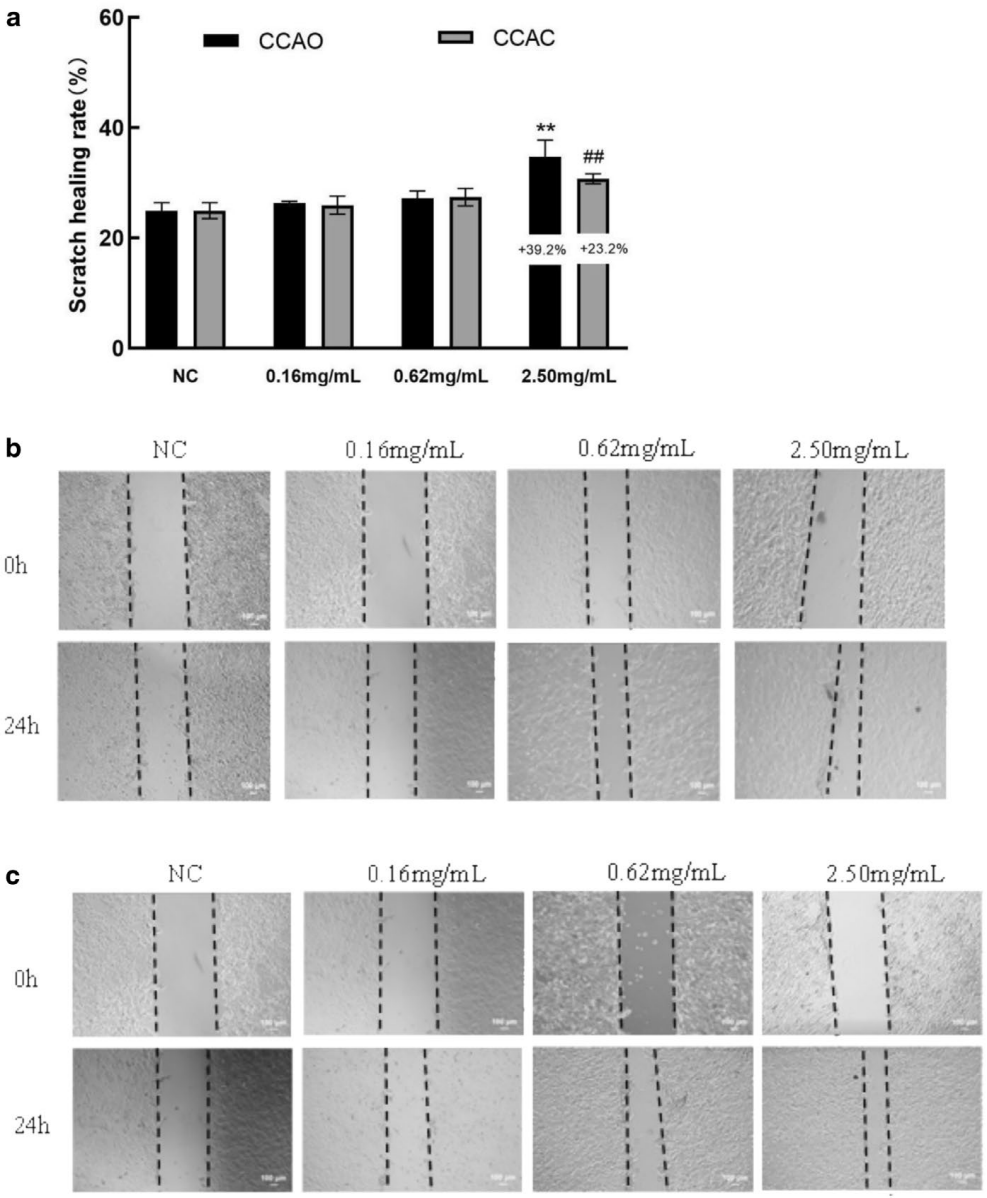
Effects of CCAC and CCAO on HaCaT cell migration. A scratch assay was used to calculate the scratch healing rate and assess the migratory ability of HaCaT cells. HaCaT cells were treated with CCAC or CCAO (0.16, 0.62, or 2.50 mg/mL, 24 h). (a) Wound healing rate. (b, c) Pictures of wound healing at 0 and 24 h time points. Magnification, ×10. The data represent three independent experiments, and the results are presented as the means ± SDs. **p < 0.01 compared with the control under CCAO treatment conditions; ##*p* < 0.01 compared with the control under CCAC treatment conditions.

The migration of HaCaT cells in response to CCAO and CCAC was measured using scratch wound assays. As presented in Figure 3, treatment with CCAO and CCAC promoted cell migration in a dose‐dependent manner. There was no significant difference in the scratch area after treatment of HaCaT cells with 0.16 or 0.62 mg/mL CCAO or CCAC for 24 h compared with the control group. Treatment of HaCaT cells with 2.50 mg/mL CCAO or CCAC for 24 h significantly reduced the scratch area by 39.22% and 23.17% (*p* < 0.05), respectively.

### 3.4 Effects of CCAC and CCAO on Barrier Maintenance‐Associated Molecules in HaCaT Cells

**FIGURE 4:**
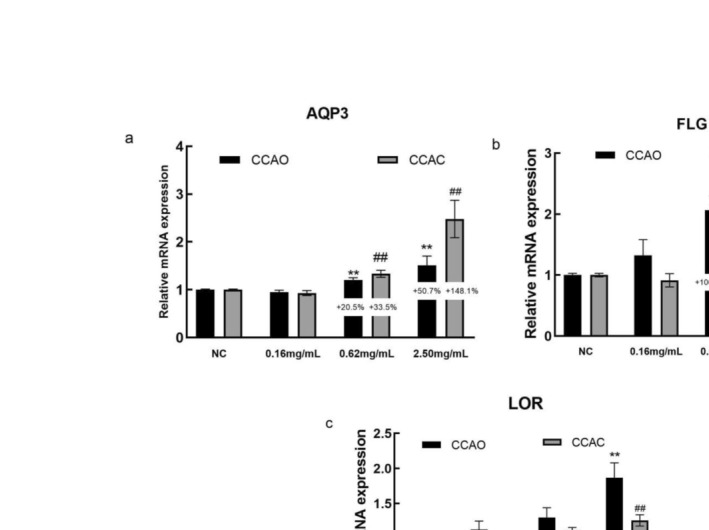
Effect of CCAO and CCAC on the expression of physical barrier‐associated molecules in HaCaT cells by RT–PCR. HaCaT cells were treated with 0.16, 0.62, or 2.50 mg/mL CCAO or CCAC for 12 h. (a–c) Skin physical barrier gene‐related AQP3, FLG, and LOR mRNA expression levels. The data represent three independent experiments, and the results are presented as the means ± SDs. *p < 0.05, **p < 0.01 compared with the control under the CCAO treatment condition; #*p* < 0.05, ##*p* < 0.01 compared with the control under the CCAC treatment condition.

**FIGURE 5:**
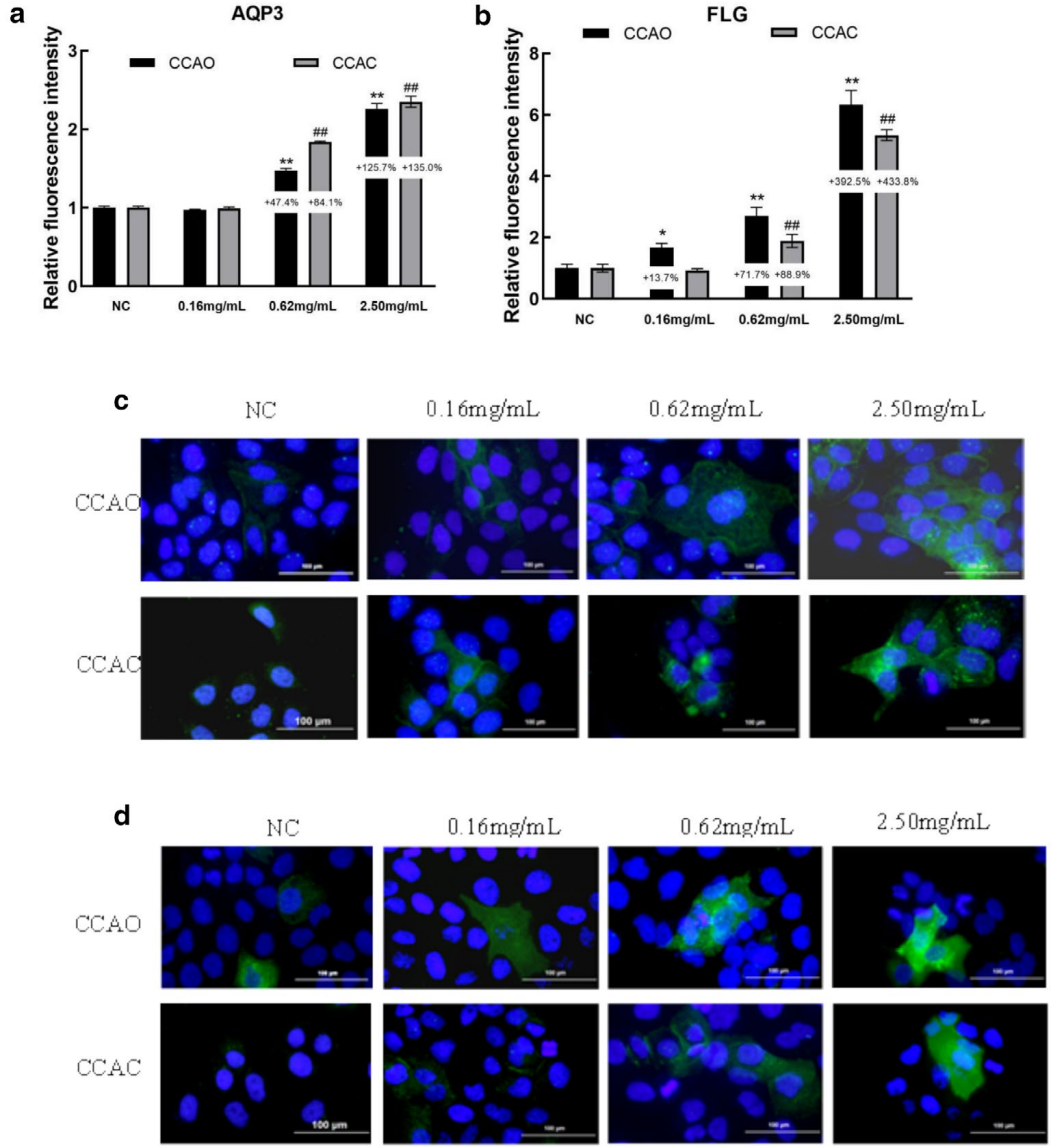
The effects of CCAO and CCAC on the expression of molecules related to physical barriers in HaCaT cells were assessed via an immunofluorescence assay. HaCaT cells were exposed to 0.16, 0.62, or 2.50 mg/mL CCAO or CCAC for 24 h. (a, b) Expression levels of the skin barrier molecules AQP3 and the FLG protein. (c, d) Fluorescence images of AQP3 and FLG distributions. Magnification, ×80. The data represent three independent experiments, and the results are presented as the means ± SDs. *p < 0.05, **p < 0.01 compared with the control under the CCAO treatment condition; #*p* < 0.05, ##*p* < 0.01 compared with the control under the CCAC treatment condition.

The filaggrin (FLG), loricrin (LOR), and aquaporin 3 (AQP3) genes are important for maintaining skin barrier function. The mRNA expression levels of the AQP3, FLG, and LOR genes increased in a dose‐dependent manner following CCAO and CCAC treatment at various concentrations (Figure 4). The protein expression of AQP3 and FLG in HaCaT cells was also determined. CCAO and CCAC treatment also dose‐dependently increased the AQP3 and FLG protein levels (Figure 5). Compared with no treatment, 2.50 mg/mL CCAO promoted FLG and LOR mRNA expression by 146.70% and 87.01%, respectively, which was significantly greater than the increases observed in the cells treated with CCAC. In addition, 2.50 mg/mL CCAC promoted AQP3 mRNA expression by 148.10%, which was significantly greater than the increase in the cells treated with CCAO. Immunofluorescence assays also revealed that CCAO had a greater effect on FLG expression than did CCAC and CCAC had a greater effect on AQP3 than CCAO did. Therefore, CCAO promoted the expression of FLG and LOR more effectively, whereas CCAC promoted the expression of AQP3 more effectively.

### 3.5 Effects of CCAC and CCAO on Antiwrinkle‐ and Firmness‐Associated Molecules in HaCaT Cells

**FIGURE 6:**
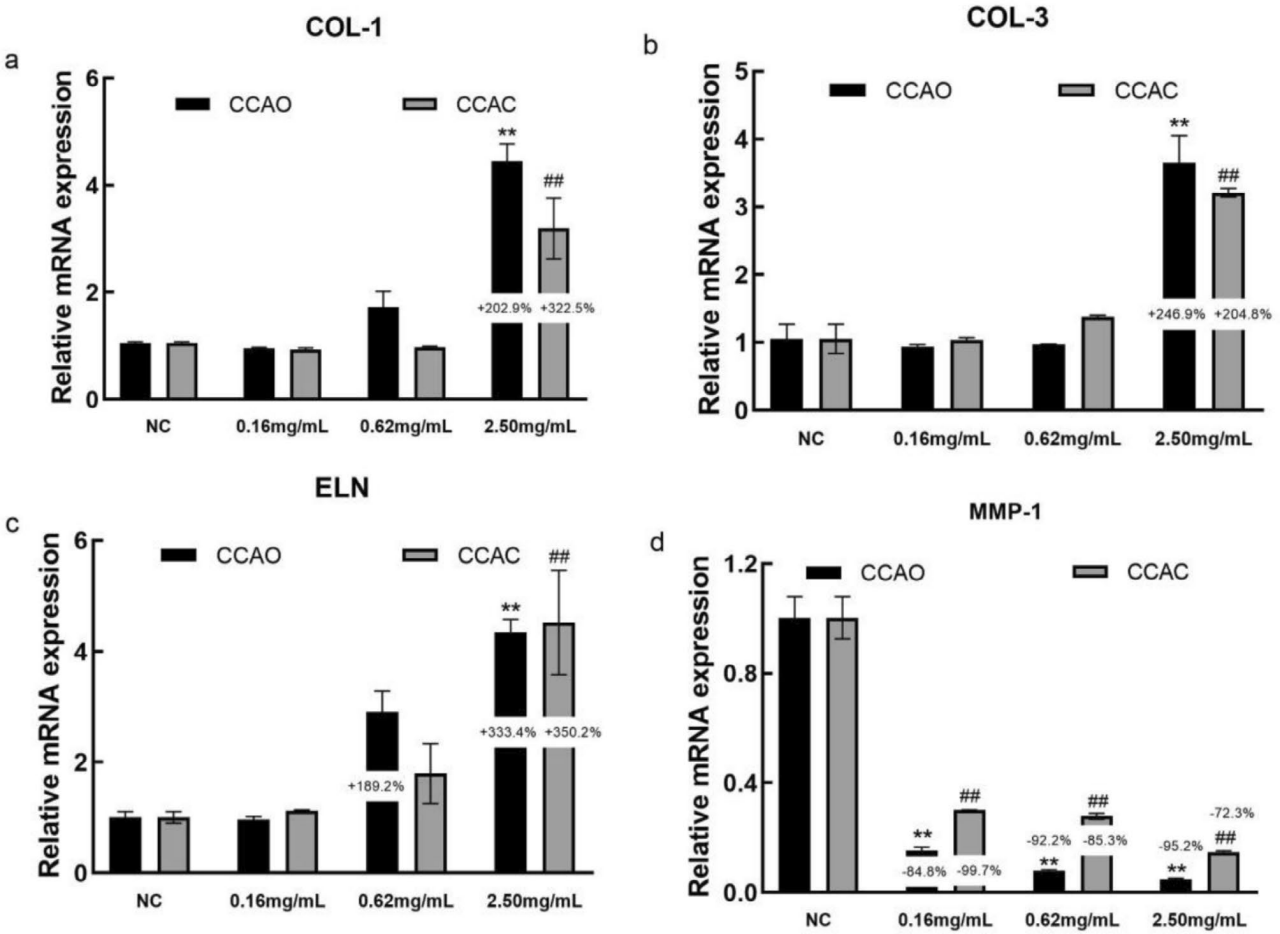
Effect of CCAO and CCAC on the expression of antiwrinkle‐ and firming‐associated molecules in HSF cells by RT–PCR. HSF cells were treated with 0.16, 0.62, or 2.50 mg/mL CCAO or CCAC for 12 h. (a–d) COL‐1, COL‐3, ELN, and MMP‐1 mRNA expression levels. The data represent three independent experiments, and the results are presented as the means ± SDs. **p < 0.01 compared with the control under CCAO treatment conditions; ##*p* < 0.01 compared with the control under CCAC treatment conditions.

**FIGURE 7:**
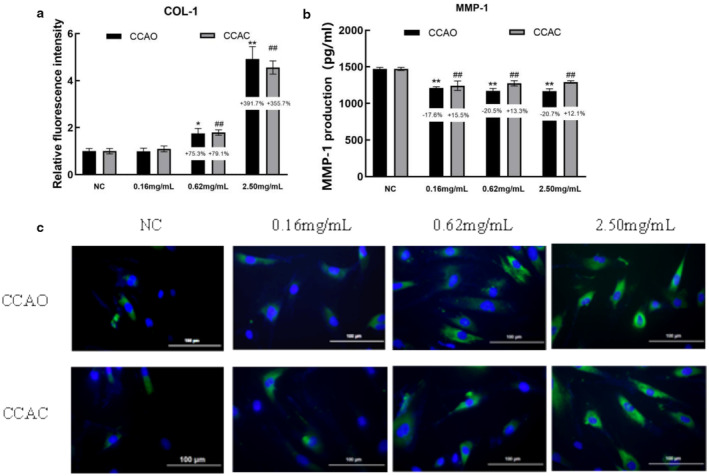
Effects of CCAO and CCAC on the expression of physical barrier‐associated molecules in HSF cells, as determined by immunofluorescence and ELISA. HSF cells were treated with 0.16, 0.62, or 2.50 mg/mL CCAO or CCAC for 24 h. (a) COL‐1 protein expression level. (b) MMP‐1 protein expression level. (c) COL‐1 distribution fluorescence image. Magnification, ×40. The data represent three independent experiments, and the results are presented as the means ± SDs. *p < 0.05, **p < 0.01 compared with the control under the CCAO treatment condition; #*p* < 0.05, ##*p* < 0.01 compared with the control under the CCAC treatment condition.

The type I collagen (COL‐1), type III collagen (COL‐3), elastin (ELN) and human matrix metalloproteinase 1 (MMP‐1) genes are important for maintaining wrinkle resistance and firm skin. The results revealed that the mRNA expression of MMP‐1 in HSF cells was significantly reduced after treatment with different concentrations of CCAO or CCAC, and the expression levels of COL‐1, COL‐3, and ELN were significantly increased only after treatment with 2.50 mg/mL CCAO or CCAC Figure 6. Moreover, compared with no treatment, 2.50 mg/mL CCAO promoted COL‐1, COL‐3, and ELN mRNA expression by 202.94%, 246.91%, and 333.367%, respectively, and reduced MMP‐1 expression by 95.21%, which was significantly greater than the effects observed with CCAC treatment (Figure 6). The expression of COL‐1 and MMP‐1 protein in HSF cells was determined as well. CCAC and CCAO treatment also dose‐dependently augmented COL‐1 protein levels and decreased MMP‐1 (Figure 7). Moreover, compared with no treatment, 0.62 and 2.50 mg/mL CCAO promoted COL‐1 protein expression by 75.3% and 391.7%, and CCAC by 79.1% and 355.7%. Three concentrations of CCAO reduced MMP‐1 expression by 17.6%, 20.5%, 20,7% and CCAC by 15.5%,13.3%,12.1% after treatment of cells, respectively. These results suggested that CCAC and CCAO might delay skin aging by upregulating the expression of antiwrinkle and firming molecules in HSF cells, and the effect of CCAO was better.

## 4 Discussion

Natural products have potentially promising nutritional and medicinal value with regard to delaying skin aging because of their multiple targets, multiple pathways, and few side effects [15, 16]. As a natural product with significant antiaging effects, CCA has been widely used in the food industry but has not been reported in the skincare industry. In addition, there has been a growing interest in recent years in using bioactive peptides to promote the production of collagen and elastin in the skin [17]. Therefore, we evaluated the role of CCA in anti‐skin aging, investigated the effects on barrier enhancement and the promotion of wrinkle resistance and skin tightening, and compared the differences in efficacy between CCAO and CCAC.

The stratum corneum is the outermost layer of the skin and is crucial for forming the skin's protective barrier [18, 19]. As we age, the stratum corneum becomes damaged, and the proliferation of keratinocytes decreases, resulting in a decrease in the barrier function of the skin, as well as a decrease in the water content within the stratum corneum [20]. In this study, CCAO and CCAC at a concentration of 2.50 mg/mL significantly promoted the proliferation and migration of HaCaT cells. In a previous study, Xiao et al. [5] have reported that CCAD significantly increased the cell viability of H_2_O_2_‐injured human gingival fibroblasts (HGFs) and reduced the cell scratch area, confirming the reparative effect of CCAD on HGF cell injury. In addition, Woonnoi et al. [10] confirmed the effect of another animal protein, hydrolyzed collagen from salmon skin, on stratum corneum repair by promoting the proliferation and migration of HaCaT cells. Furthermore, barrier damage affects the expression of the barrier‐related molecules AQP3, FLG and LOR [21]. In this study, both CCAO and CCAC at a concentration of 2.50 mg/mL significantly promoted the expression of AQP3, FLG, and LOR; CCAO had a greater effect on FLG and LOR, whereas CCAC promoted the expression of AQP3. Previous studies have demonstrated that collagen hydrolysate can effectively improve disordered AQP3 expression in UVB‐damaged HaCaT cells, restore FLG expression, and repair UVB‐induced damage to the skin barrier in mice [22]. The differences in the effects of CCAO versus those of CCAC may be related to the bioavailability of CCAO and CCAC. CCAO has high bioavailability because of its small molecular weight, stable structure, and strong activity level [23, 24]. CCAO is more likely to enter the cell [25], thus affecting the expression of the intramembrane proteins FLG and LOR, increasing the likelihood of promoting the formation of the skin barrier [26]. In contrast, CCAC has a relatively high molecular weight, and the effective components, therefore, have a more significant effect on the membrane‐localized protein AQP3, making it more likely to influence skin water and glycerol transportation [27].

Decreases in collagen synthesis, wrinkle formation, and decreased skin elasticity are common features of skin aging. Type I collagen (COL1) plays a role in the formation of collagen fibers [28]. Type III collagen (COL3) is found between the epidermis and the dermis and is a key component that supports the epidermis [29]. Matrix metalloproteinase‐1 (MMP‐1) is the most important enzyme involved in the degradation of COL‐I/III [30]. Elastin (ELN) is an extracellular matrix protein expressed in the dermis of the skin [31]. Therefore, the expression of COL‐1, COL‐3, ELN, and MMP‐1 is closely related to skin aging. In this study, CCAO and CCAC significantly upregulated the expression of COL‐1, COL‐3, and ELN and downregulated the expression of MMP‐1. CCAO was more effective. Owing to the significant antiaging effects of CCA, there have been many reports on the antiwrinkle and firming effects of CCA. Xiao et al. [5] found that CCAD could suppress the UVA‐induced reduction in the expression of type IV collagen and elastin in both HGFs and 3D skin equivalents, suggesting that CCAD can effectively mitigate UVA‐induced skin aging. Qin et al. [32] The effects of three different molecular weights of marine fish skin collagen oligopeptide on UVB‐induced photoaging in rat skin and the results revealed that the medium molecular weight marine fish skin collagen oligopeptide had the most significant effects on the recovery of UVB‐damaged skin tissue and the promotion of collagen and elastin. These findings suggest that lowering the molecular weight of a protein to a certain extent can make it more effective against aging. The findings of these studies are in accordance with our findings. We showed that the small molecule CCAO has strong biological activity and, compared with CCAC, has greater antiaging effects.

## 5 Conclusions

In conclusion, the local application of CCA can improve the skin barrier, increase the skin collagen content, and increase skin elasticity, thereby delaying skin aging. These effects are mediated by the regulation of the expression of barrier‐ and aging‐related genes and proteins. The antiaging effects of CCAO are slightly greater than those of CCAC, which might be due to its lower molecular weight and greater bioactivity, so their skin permeation and bioactivity should be further verified. Subsequently, the preparation process of CCA should be improved. In this study, the effects of CCAO and CCAC in delaying skin aging were investigated only at the cellular level, and their mechanism of action and human trials are still to be explored. This study provides a solid scientific basis and theoretical support for the skin aging‐delaying effects of CCA, which can help to broaden the scope of application of traditional medicinal and foodstuffs in the cosmetic and health food industry.

## Author Contributions

Fan Wu, Xiao Xie and Yao Pan designed the study. Fan Wu performed the experiments and analyzed the data. Fan Wu and Yao Pan prepared the manuscript. Xiao Xie, Wenwen Ru, Xiaofei Guo, Lingfeng Meng, Jianling Zhang and Xiuling Liu provided the samples and contributed to the interpretation of the results. All authors have reviewed the manuscript.

## Conflicts of Interest

Xiao Xie, Wenwen Ru, Xiaofei Guo, Lingfeng Meng, Jianling Zhang and Xiuling Liu are employees of the Dong‐E‐E‐Jiao Co. Ltd. Dong‐E‐E‐Jiao Co. Ltd. develops and produces the CCAO and CCAC. Other authors declare no conflicts of interest.

## Data Availability

The data that support the findings of this study are available from the corresponding author upon reasonable request.
